# Men’s Self-Reported and Psychophysiological Affective Responses to Sexual Violence and Their Associations with Rape Myths, Personality, and Sexual Traits: A Preliminary Study

**DOI:** 10.1080/19317611.2023.2200786

**Published:** 2023-05-04

**Authors:** Joana Carvalho, Pedro J. Rosa, Erick Janssen

**Affiliations:** a Department of Education and Psychology, William James Center for Research, University of Aveiro, Aveiro, Portugal; b Center for Psychology, University of Porto, Porto, Portugal; c Digital Human-Environment Interaction Lab (HEI-Lab), Lusófona University, Lisbon, Portugal; d ISMAT, Transdisciplinary Research Center (ISHIP), Portimão, Portugal; e Department of Neurosciences, Institute for Family and Sexuality Studies, University of Leuven, Leuven, Belgium

**Keywords:** Emotions, sexual violence, sexual arousal, psychophysiology, fEMG, EDA, rape myths, psychopathy, personality

## Abstract

*Objective:* Despite alarming evidence on sexual violence against women, little is known about men’s emotional responses to rape and how these may be involved in sexual violence dynamics. Accordingly, our aim was to capture how rape scenarios are emotionally appraised. *Methods:* The current study evaluated men’s (N = 30) self-reported and psychophysiological emotional responses (facial EMG, electrodermal activity) to a rape scene, and contrasted it with their responses to stimuli depicting nonsexual violence and nonviolent male-female interactions. The associations between men’s emotional responses and their endorsement of rape myths, personality, and sexual traits were also examined. *Results:* Findings revealed that the rape scene resulted in higher negative affect, both subjectively and indexed by increased facial EMG (corrugator activity), than the other two stimuli. Additionally, personality traits of neuroticism, lower agreeableness, lower consciousness, psychopathic tendencies, as well as lower sexual inhibition proneness, were all associated with higher subjective sexual arousal toward rape. *Conclusions:* Findings add to the literature on the putative emotional processes underpinning the appraisal of sexual violence against women.

Sexual violence in the context of intimate relationships, human trafficking, or (acquaintance) rape affects women disproportionately (World Health Organization, 2013). According to the European Union Agency for Fundamental Rights (2014), 1 in 10 women in the EU have been a victim of sexual violence and 1 in 20 have been raped (European Union Agency for Fundamental Rights, 2015). In the US, it has been estimated that close to 45% of women have experienced some form of sexual violence (Smith et al., 2018). Prevalence rates in non-western countries are compounded by additional forms of sexual violence (e.g., gang rape tactics of political repression; Vahedi et al., 2021), also mostly perpetrated by men and directed toward women.

From a legal perspective, attrition, or the loss of cases within the criminal justice system from police to conviction level, is particularly high for sexual crimes (Jordan, 2011). This finding points at a leniency bias toward those who commit sexual offenses (Bieneck & Krahé, 2011), raising questions about how people, in the legal system and beyond, appraise and respond to sexual violence of women (Hansen et al., 2015; Morabito et al., 2019). In this way, rape myths, that is, stereotyped or false beliefs about rape (Lonsway & Fitzgerald, 1994), establish the nature of sexual offending behavior, legitimating sexual assault (Walfield, 2021). While rape myths are disseminated across the general population as well as among justice and healthcare professionals (Anderson & Quinn, 2009; Kassing & Prieto, 2003; Turchik & Edwards, 2012), in general, a robust gender difference exists, with en endorsing more rape myths than women (Rosenstein, 2015; Walfield, 2021). Rape myths support sexual violence of women and influence how individuals appraise and respond to sexual violence situations, including its victims. Rape myths usually concern the blaming of the victim (e.g., women as the trigger of the offense because of bold behavior, clothing), disclosure credibility (e.g., women searching for revenge), justification of offender’s behavior (e.g., being passionate), context (e.g., sex workers are not a target of rape), or impact (e.g., no real damage; women can simply proceed with their lives) (Beshers & DiVita, 2021; Smith & Skinner, 2017). Rape myths constitute a form of cultural appraisal that shapes how sexual violence is approached; for example, rape myths are believed to influence the course of trials favoring those who offend (Smith & Skinner, 2017).

The cognitive appraisal (e.g., rape myths) of sexual offending behavior has been the topic of systematic research. However, little is known about the *affective* appraisal of sexual violence, both in terms of self-reported emotions and psychophysiological reactions. Past studies have addressed the emotional responses to violent scenes in order to understand the psychophysiological mechanisms underpinning human aggressive behavior or its acceptance. While some of these studies have targeted the emotional responses of individuals when exposed to scenes displaying physical violence against a human target (i.e., beating, stabbing, shooting; e.g., Fanti et al., 2015, 2017), only a few have considered the specificities of sexual violence and women as targets of violence. For example, Carvalho and Rosa (2020) found that men reported more positive emotions toward a rape film than women, but no differences were found in other emotional and psychophysiological (e.g., pupil dilatation) responses. Interestingly, pupil dilation decreased throughout the course of the film clip suggesting that individuals’ affective reactions may eventually adapt in the context of rape. Indeed, depictions of sexual violence to women, suggest that rape scenarios, overall, do not inhibit sexual arousal (Malamuth et al., 1986; Malamuth & Check, 1980).

In addition, a few studies have examined predictors of individual variability in sexual and affective responses to sexually violent stimuli. For example, an increased likelihood of perpetrating rape, aggressive personality traits, and powerful/aggressive sexual self-schemas have all been found to be associated with men’s genital and reported sexual arousal to rape themes (Carvalho, & Rosa, 2020; Malamuth, & Check, 1983). Sexual traits, including the propensities for sexual excitation and sexual inhibition, have been also considered. Sexual inhibition, especially inhibition due to possible negative consequences of being sexually aroused, is predictive of men’s genital responses to films depicting sexual coercion. In addition, a lower propensity for sexual inhibition is associated with self-reported perpetration of sexual aggressive behaviors (Carvalho, et al., 2013; Peterson et al. 2010).

Emotional responses to most stimuli and stimulus events are rooted in an individual’s cognitive appraisal of such an event (Smith & Kirby, 2001), in some cases leading to a corresponding pattern of physiological arousal (Tomaka et al., 1993). Emotional response to a stimulus may highlight core mechanisms underpinning individuals’ behavior such as decision-making regarding that stimulus situation (Lerner et al., 2015). Furthermore, emotions drive us toward goal-oriented actions, preventing harmful or negative social interactions, or promoting pleasant and positive social interactions (Lang et al., 1997; Mirabella, 2018). Accordingly, research on emotional responses to sexual violence toward women may contribute to our understanding of individual and social responses to and mechanisms underlying sexual violence. In view of and consistent with this, the aim of the current study was to assess men’s subjective and psychophysiological emotional responses to a sexually violent scene, and to compare them to responses to a non-sexual violent scene.

Beyond rape myths and sexual traits, personality traits have been found to contribute to sexual violence dynamics as well. Neuroticism and low conscientiousness and agreeableness have all been related to self-reported use of sexually coercive strategies (Carvalho et al., 2021; Carvalho & Nobre, 2019; Voller & Long, 2010). Furthermore, lower agreeableness has been found to predict attitudes supporting sexual violence of women (Hald & Malamuth, 2015). Despite their more general association with offending behavior, associations between psychopathic traits and sexual offending have been less consistent (Jolliffe & Farrington, 2004; Knight & Guay, 2018). Still, psychopathic traits, even in community samples, have been found to be predictive of more positive and less negative emotional responses to nonsexual violent scenes and of less sympathy toward victims (Fanti, Kyranides, & Panayiotou, 2017; Fanti, Kyranides, Georgiou, et al., 2017). Accordingly, some of these traits may be involved in the emotional processing of sexual violence and relevant to the mechanisms underlying individuals’ appraisal of and actions in contexts of sexual violence.

The current study examined men’s emotional responses to a film clip depicting the rape of a woman. Responses included self-reported positive and negative affect, as well as the experience of sexual arousal, and psychophysiological affective activation as indexed through the measurement of facial electromyography (fEMG) and electrodermal activity (EDA). To control for possible nonspecific effects of violent content, we compared responses to a sexually violent clip with responses to a film clip depicting nonsexual physical violence, also involving a woman, and a control film clip depicting a nonviolent male-female social interaction. We also evaluated associations among subjective and psychophysiological responses to the rape scene. In addition, to explore possible individual differences in rape responses, we examined associations between emotional responses to rape, personality (including psychopathic tendencies), sexual traits and the endorsement of rape myths. On the basis of the existing empirical literature, we formulated the following hypotheses: 1) As compared to nonsexual violent scenes, depictions of rape will induce 1) more sexual arousal; 2) less negative affect and more positive affect (self-report, fEMG), 3) stronger psychophysiological activation (EDA), and 4) personality traits of neuroticism, conscientiousness, and agreeableness; psychopathic tendencies; and lower propensity for sexual inhibition will be associated with affective and sexual responses to the rape scene.

## Methods

### Participants

A young, educated sample of 30 men^1^ agreed to participate in this experiment. Inclusion criteria were being heterosexual and age ≥18 years. Participants’ mean age was 29.6 (SD = 9.38), ranging from 18 to 50 years; 90% were single, 10% were married, and most had a university degree (80%). The study was advertised through the Internet (Facebook and institutional university email). Participants were advised on the content of the video-clips prior to participation. The study was conducted after written informed consent, and no compensation was given. This study was reviewed and approved by the local ethics committee at Universidade Lusófona, in Lisbon, Portugal. After the experiment, participants were debriefed for emotional and physical discomfort; no such discomfort was reported, and all participants completed the session.

### Procedure and stimuli

At arrival in the laboratory, participants received a detailed description of the procedures, provided informed consent, and completed a set of self-report measures, including demographics. Following this, participants were escorted to the experimental room for the placement of EDA and fEMG electrodes. Participants were exposed to three 40-second video clips, in counterbalanced order: 1) rape of a woman by a man, 2) nonsexual, physical violence toward a woman, perpetrated by man, and 3) nonsexual, friendly social interaction between a man and a woman. All three clips were taken from commercially available films. In order to provide a baseline for the psychophysiological metrics, participants were first exposed to a neutral clip, which was taken from a nature documentary. A pilot test in an independent sample of 32 men revealed that the two violent films did not differ in terms of perceived violence (*p* > .05) and intensity of violence (*p* > .05), and both scored higher in the presence and intensity of violence than the nonviolent social interaction clip (all *p*s < .001). Following the violent and nonviolent clips, participants rated their emotions and subjective sexual arousal (described below). Film stimuli and questions were presented with Tobii Studio v 3.3.2 software (Tobii Technology AB, Sweeden) on a TFT 17’’ monitor. EDA and fEMG signals were recorded continuously throughout the session.

### Measures

Rape Myths Scale (RMS; Martins et al., 2012). This is a 30-item self-report scale measuring the endorsement of stereotypical/prejudicial beliefs about rape (e.g., rape requires physical violence, victim’s behavior was appealing). The original/Portuguese version of the scale supported its validity and reliability (Martins et al., 2012). In the current study, the Cronbach alpha was .90.

Five-Factor Inventory (NEO-FFI; Costa & McCrae, 1992). The NEO-FFI is a 60-item self-report scale assessing five personality domains: neuroticism (the tendency toward negative affect, impulsiveness, poor coping skills), extroversion (the tendency toward sociability, proactivity, assertiveness), openness (to be open to new experiences, accepting novelty), agreeableness (the tendency to be kind, trusting), and conscientiousness (characterized by moral standards; prudent/goal-oriented individuals). Both the original and Portuguese versions of the scale supported validity and reliability (Costa & McCrae, 1992; Magalhães et al., 2013). In the present study, Cronbach alphas ranged from .78 (openness) to .88 (conscientiousness).

Youth Psychopathic Traits Inventory – Short Version (YPI-S; Van Baardewijk et al., 2010). The YPI-S is an 18-item self-report scale assessing psychopathic traits according to three dimensions: grandiosity-manipulative (e.g., good at manipulating people), callous-unemotional (e.g., reduced emotional tone or empathy), and impulsive- irresponsible (e.g., impulsive, reckless). The psychometric properties have been established regarding the original and Portuguese versions (Pechorro et al., 2015, 2017; Van Baardewijk et al., 2010). The scale has been further used with adult samples, suggesting validity and reliability (e.g., Colins & Andershed, 2016). In the current study, the Cronbach alpha ranged from .70 (impulsive- irresponsible) to .86 (grandiosity-manipulative).

Sexual Inhibition/Excitation Scales (Janssen, et al. 2002). This questionnaire consists of 45 items and assesses the propensity for sexual excitation (SES, e.g., getting easily aroused), sexual inhibition due to the threat of sexual performance failure (SIS1, e.g., fearing losing erection), and sexual inhibition due to the threat of sexual performance consequences (SIS2, e.g., losing arousal if there is a risk of catching a sexually transmitted disease). The scale revealed validity and reliability, both in the original (Janssen et al., 2002) and the Portuguese version (Quinta-Gomes et al., 2018). In the present study, Cronbach alphas ranged from acceptable to excellent (SES = .91, SIS1 = .82, SIS2 = .76).

Positive and Negative Affect Schedule (state form) (Watson et al., 1988). This is a 20-item self-report measure targeting positive (e.g., interested, excited) and negative (e.g., distressed, frightened) affect. In the current study, participants responded the state version, i.e., how much did they feel these emotions immediately after watching the film-clips. This measure presents validity and reliability in the original (Watson et al., 1988) and Portuguese version (Galinha & Pais-Ribeiro, 2012). In the current study, the Cronbach alphas for each film clip were as follows: Positive affect/rape clip = .75, negative affect/rape clip = .92; positive affect/physical violence clip = .92, negative affect/physical violence clip = .94; and .96 to both positive and negative affect to the normal/nonviolent social interaction film clip.

Subjective Sexual Arousal (Carvalho et al., 2017). This previously used set of items assesses subjective sexual arousal to film clips. Participants are asked to rate their level of sexual arousal, erection, and desire to engage in sex (with someone else or alone) using Likert scales that range from 0 “not at all” to 9 “extremely,” and combined into a single score. In the current sample, the Cronbach alphas were as follows: Rape clip = .95, physical violence clip = .99, normal/nonviolent social interaction clip = .94.

### Psychophysiological measures

**Figure 1.**
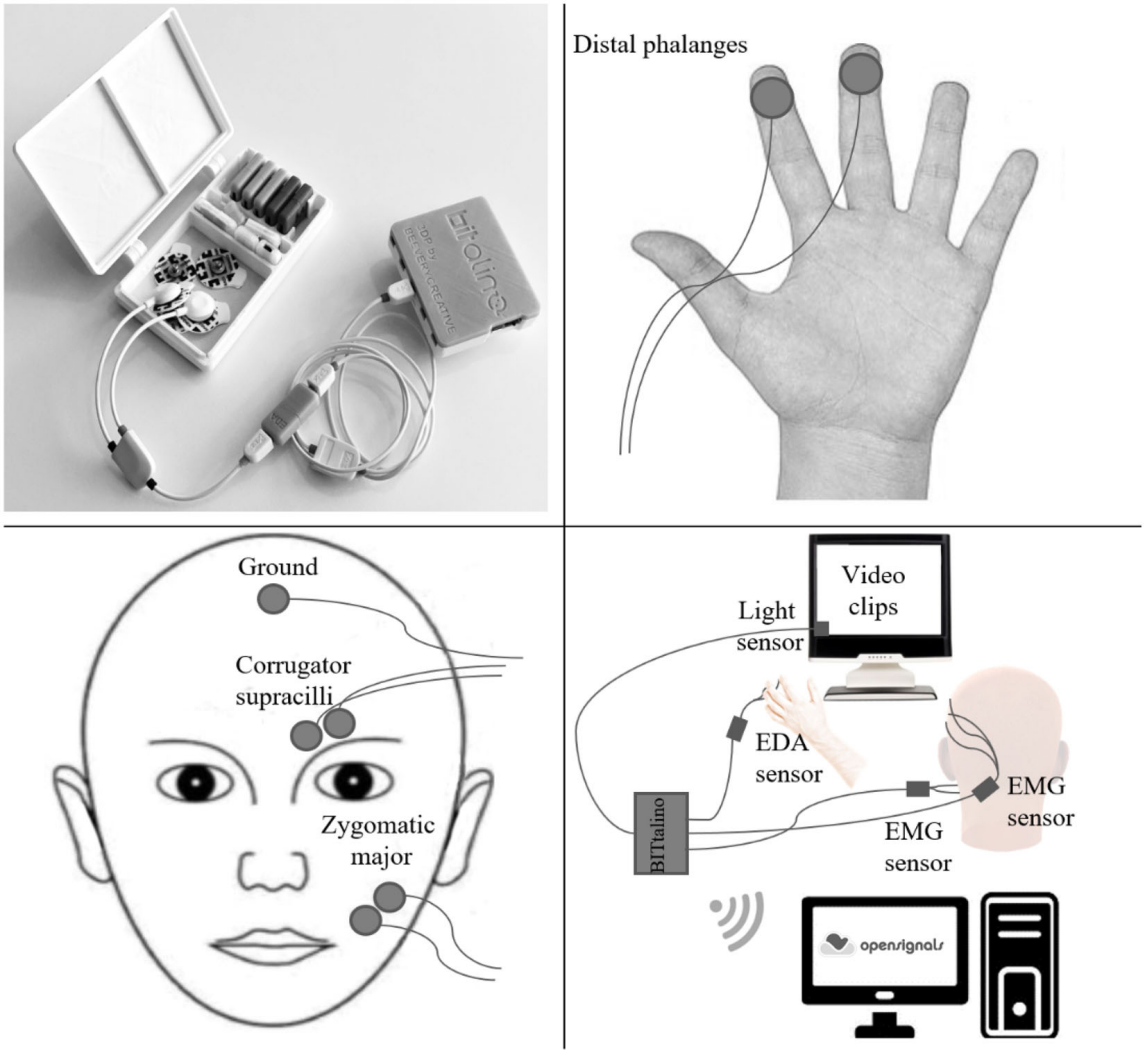
Physiological recording equipment, electrodes placement, and general laboratory setup.

EDA and fEMG signals were acquired using BITalino (Plux Wireless BioSignals SA), a portable biosignal acquisition system, and OpenSignals software, both running on a desktop computer. While EDA functions as a marker of emotional arousal and autonomic activation (Rosa et al., 2017), fEMG can be used to measure negative (corrugator activity) and positive (zygomaticus activity) valence (Cacioppo et al., 1986; Larsen et al., 2003). EDA was measured using 11 mm silver/silver chloride (Ag/AgCl) pre-gelled and self-adhesive disposable electrodes, placed on the distal phalanges of the index and middle fingers of the non-dominant hand. With regards to the fEMG signal, 8 mm bipolar silver/silver chloride (Ag/AgCl) pre-gelled and self-adhesive surface disposable electrodes with a bipolar configuration were positioned parallel to participants’ left corrugator supercilii and zygomaticus major muscle fiber regions as depicted in Figure 1.

The fEMG montage followed the recommendations of Fridlund and Cacioppo (1986). Both EDA and fEMG signals were recorded at a sampling rate of 1000 Hz. A light sensor was placed at the upper left corner of the display for marking the video-clip onset via OpenSignals revolution (Plux) acquisition software (see Figure 1).

### Physiological data reduction

Prior to analysis, signal artifacts were identified and excluded. Raw EDA data were down-sampled to 200 Hz time resolution and filtered with a second-order Butterworth low-pass filter with a cutoff frequency at 5 Hz. Changes in Skin Conductance Level (SCL) related to tonic sympathetic activation were analyzed (Boucsein, 2012). The SCL ratio was computed as the mean SCL value of the 40s following each video-clip onset divided by the baseline mean SCL (−2s). In order to minimize interindividual variability, the SCL ratio was also range-corrected according to the recommendations of Lykken and Venables (1971). With regards to fEMG, the raw signal was filtered using a band-pass 4th-order Butterworth filter with cutoff frequencies set at 28 Hz and 250 Hz and then smoothed with a 100 ms moving average. The fEMG ratio was computed just like the SCL ratio. All physiological data were processed using Acqknowledge 4.1 (Biopac Systems).

### Statistical approach

Repeated measures ANOVAs with Huynh-Feld correction were performed to test the main effects of stimulus condition on the subjective and psychophysiological responses; multiple comparisons were considered after Bonferroni adjustment. IBM-SPSS Statistics (version 25), was used for this set of analysis. *Effect sizes were interpreted according to Cohen’s* (1988) guidelines. Pearson product-moment correlations, with Holm’s correction for multiple comparisons, were done to examine associations between rape myths, personality and sexual traits, and the subjective/psychophysiological responses to the sexually violent clip, and to test the associations between self-reported emotions and psychophysiological metrics. These last analyses were conducted using Package “psych” (v 2.2.9; Revelle, 2022) for R software (R Core Team, 2022). The statistical significance was set at *p* < .05.

## Results

### Subjective affective responses

**Figure 2.**
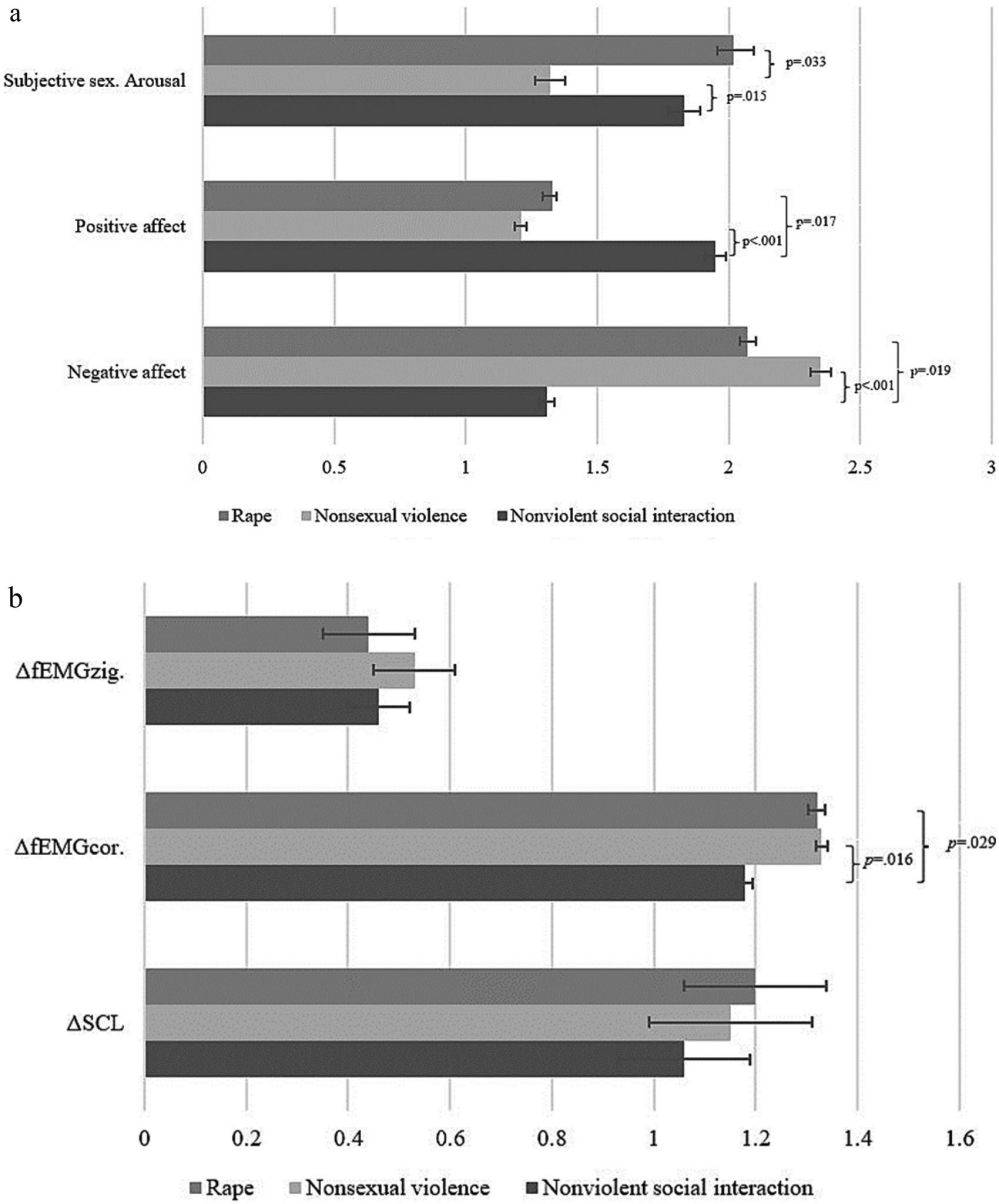
(a) Pairwise comparisons of sexual arousal and self-reported positive and negative affect. (b) Pairwise comparisons of zygomatic EMG, corrugator EMG, and EDA.

For all three variables (positive and negative affect, and subjective sexual arousal) a significant main effect of film was found (positive affect, *F* (1.73, 50.047) = 10.445, *p* < .001, partial *η*^2^ = .27; negative affect, *F* (1.68, 48.715) = 12.482, *p* < .001, partial *η*^2^ = .30; subjective sexual arousal, *F* (1.69, 48.974) = 4.727, *p* = .018, partial *η*^2^ = .14). The nonviolent social interaction resulted in significantly more positive affect (M_nonviolent_ = 1.95, SE = .20; M_rape_ = 1.33, SE = .08, Cohen’s *d* = 2.26; M_physical violence_ = 1.21, SE = .12, Cohen’s d = 2.48), and less negative affect (M_nonviolent_ = 1.31, SD = .15; M_rape_ = 2.08, SE = .18, Cohen’s *d* = .59; M_physical violence_ = 2.53, SE = .21, Cohen’s *d* = 3.70) than the sexual and nonsexual violence scenes. No differences were found between the two violent scenes (see Figure 2a). As for sexual arousal, although no significant differences were found between the rape and nonviolent interaction scene, both of these scenes resulted in significantly more subjective sexual arousal than the nonsexual violent scene (M_nonviolent_ = 1.83, SE = .33, Cohen’s *d* = .91; M_rape_ = 2.02, SE = .39, Cohen’s *d* = 1.81; M_physical violence_ = 1.32, SE = .30). See Figure 2a.

### Psychophysiological responses

Findings on the psychophysiological responses revealed a significant main effect of film on corrugator responses, indexing negative valence, *F* (2, 50) = 3.212, *p* = .049, partial *η*^2^ = .11. The rape scene and the nonsexual violent scene resulted in significantly stronger corrugator responses compared to the nonviolent social interaction scene (M_nonviolent_ = 1.19, SE = .06; M_rape_ = 1.32, SE = .09, Cohen’s *d* = .49; M_physical violence_ = 1.33, SE = .08, Cohen’s *d* = .56). No significant main effects were found for zygomatic EMG activity, *F* (1.66, 48.066) = 1.319, *p* = .273, partial *η*^2^ = 04 nor EDA activity, *F* (2.00, 54.000) = .542, *p* = .585, partial *η*^2^ = 02; see Figure 2b).

#### Associations between emotional responses to rape and the endorsement of rape myths, personality, and sexual traits

Correlations between participants’ emotional responses to rape and self-reported rape myths, personality and sexual traits are presented in Table 1. Data regarding personality traits showed that neuroticism was associated with increased subjective sexual arousal to rape (*r* = .41, *p* = .020), while agreeableness and consciousness were associated with less subjective sexual arousal (*r* = −.53, *p* < .001; *r* = −.52, *p* < .001, respectively). As for psychopathic tendencies, the grandiosity-manipulative style and the impulsive-irresponsible style were associated with increased subjective sexual arousal to rape (*r* = .55, *p* < .001; *r* = .63, *p* < .001, respectively). Finally, the propensity for sexual inhibition due to the fear of the negative consequences associated to sex, was correlated with subjective sexual arousal toward rape, as well (*r* = −.46, *p* = .010). No further significant correlations were found.

**Table 1.** Pearson product-moment correlations (with Holm’s correction) between participants’ endorsement of rape myths, personality, psychopathic, and sexual traits, and the emotional responses to the rape scene.

	Self-reported positive affect	Self-reported negative affect	Subjective sexual arousal	fEMG zygomatic	fEMG corrugator	EDA
Rape myths						
Total score	.16	−.35	.27	.03	.03	−.08
Personality traits						
Neuroticism	.24	−.12	.41*	.12	.07	−.30
Extroversion	−.01	.21	−.34	.16	.08	.05
Openness	−20	−.19	.07	−.21	.15	.06
Agreeableness	−.16	.18	−.53**	−.02	−.07	.13
Conscientiousness	−.07	.25	−.52**	−.07	−.05	.01
Psychopathic traits						
Grandiosity-manipulative	.35	.10	.55**	.26	−.02	−.04
Callous-unemotional	.21	−.00	.17	−.03	.17	.23
Impulsive-irresponsible	.30	.03	.63**	.35	.26	−.18
Sexual propensities						
SES	.04	−.14	.23	.27	−.16	−.04
SIS1	.10	−.16	−.13	.13	−.20	−.10
SIS2	−.28	−.04	−.46*	.10	-.09	.10

SES: Sexual excitation; SIS1: inhibition due to the threat of sexual performance failure; SIS2: sexual inhibition due to the threat of sexual performance consequences. *p < .05, **p < .001.

#### Associations between self-reported emotions and psychophysiological metrics

In order to verify task validity, correlations between participants’ subjective responses and psychophysiological activation were considered (see Table 2). Subjective sexual arousal to the rape scene was positively associated with zygomaticus activity (*r* = .45, *p* = .010), and negative affect to the rape scene was positively associated with corrugator activity (*r* = .41, *p* = .020).

**Table 2.** Pearson product-moment correlations (with Holm’s correction) between participants’ subjective responses and psychophysiological activation.

	Self-reported positive affect	Self-reported negative affect	Subjective sexual arousal
fEMG zygomatic	.01	−.01	.45*
fEMG corrugator	.01	.41*	−.08
EDA	−.11	.06	−.11

*p < .05.

## Discussion

In this study, we assessed men’s subjective and psychophysiological affective responses to a sexually violent scene while comparing them to responses to nonsexually violent and nonviolent social interaction scenes. In addition, we explored associations among subjective, psychophysiological responses, and key metrics for the understanding of sexual offending behavior: rape myths, personality, and sexual traits. Contrary to our expectations, participants did not report more positive and less negative affect to the rape as compared to the nonsexually violent scene. However, as predicted, men reported higher levels of subjective sexual arousal to the rape scene than to the nonsexually violent scene. Interestingly, the sexually violent stimulus seemed to have induced a specific response pattern, as the rape scene, compared to the nonviolent interaction scene, combined both higher subjective negative affect and higher levels of subjective sexual arousal. That pattern may be of particular interest to our understanding of sexual offending behavior. Indeed, past studies found that sexual film clips resulting in sexual arousal combined with negative feelings were associated with increased retaliatory aggression in an experimental setting (Zillman et al., 1981) supporting the assumption that aggression may be facilitated by the combination of sexual arousal and negative affect (Sapolsky, 1984). In addition, we believe that the similarities regarding sexual arousal levels toward the rape and non-sexual social interaction scene may be explained by the attractiveness of the male and female character in the last condition.

Contrary to our hypotheses, but in line with the findings regarding self-reported negative affect to the rape and nonsexual violent scenes, psychophysiological data revealed that these scenes induced stronger corrugator activity, i.e., negative affect, compared to the nonviolent social interaction scene. No further effects were detected. In all, while men seemed to present an avoidance response pattern regarding the nonsexual violence condition (as reflected by higher self-reported and psychophysiological negative affect and lower subjective sexual arousal), our findings are suggestive of approach tendencies toward the sexual violence scenario, as indicated by higher levels of reported sexual arousal despite increased subjective and psychophysiological negative affect. As for the absence of EDA effects, it is possible that individuals were familiar with violent contents, resulting in similar activation across film clips. An alternative explanation involves the presence of possible floor effects. However, the clips displaying violence were rated as more violent and intense and clearly departed in terms of visual content and semantics from the nonviolent social interaction scene. Subjective sexual arousal and self-reported negative affect toward the rape scene were associated with zygomaticus (positive valence) and corrugator activity (negative valence), respectively, suggesting a corresponding pattern between self-reported and psychophysiological responses, which further supports the validity of the experimental task.

In support of our last hypothesis on the associations between the emotional responses toward rape, and the endorsement of rape myths, personality, and sexual traits, findings revealed a series of associations regarding self-report metrics of emotional response. Traits of neuroticism, agreeableness, and consciousness, were associated with subjective sexual arousal toward rape. While neuroticism was associated with higher levels of arousal, agreeableness and consciousness were associated with lower rates. These are interesting findings as previous evidence on sexual offending behavior committed by young and educated community samples revealed that neuroticism, low agreeableness, and low consciousness were involved in sexual violence perpetration (Carvalho et al., 2021; Carvalho & Nobre, 2019); similarly, lower agreeableness predicted supportive attitudes of women’s sexual violence (Hald & Malamuth, 2015). The current results add to previous literature supporting the role of neuroticism, agreeableness, and consciousness in the context of sexual violence, and suggesting that sexual violence dynamics, e.g., sexual offending or appraisals of sexual violence, may share common personality traits. Furthermore, findings on psychopathic traits showed that the grandiosity-manipulative and the impulsive-irresponsible styles were associated with increased subjective sexual arousal to rape. While the role of psychopathic traits is inconsistent across the literature of sexual offending behavior (Knight & Guay, 2018), psychopathic tendencies were involved in exploitative mating strategies in men (Jonason et al., 2009). In addition, impulsiveness was associated with sexual perpetration in convicted and community male samples (Carvalho & Nobre 2013). Also, the current findings align with previous evidence showing that the grandiosity-manipulative psychopathic style was associated with higher ratings of positive affect toward violent, non-sexual scenes (Fanti, Kyranides, Georgiou, et al., 2017). There seems to be evidence suggesting that psychopathic tendencies among community samples may be involved in sexual offending dynamics, whether these include sexual offending behavior or appraisals. Finally, lower sexual inhibition due to the threat of negative consequences resulting from sex (SIS2) was associated with increased subjective sexual arousal to the rape scene. This association is consistent with previous studies showing that SIS2 is associated with sexual responses to rape films (Janssen et al., 2002) and self-reported sexual aggression in community men (Peterson et al., 2010). In all, these preliminary findings point that personality and sexual traits should be accounted for in prevention strategies regarding sexual violence of women.

The current study has several limitations. First, although the sample size was based on a power analysis that indicated it to be sufficient to detect main effects, some associations and effects may have required a larger sample size to be detected. For that reason, we must consider the preliminary nature of the current study and be cautious regarding the overinterpretation of findings. Also, it is worth noting that the average levels of subjective sexual arousal were low. These findings do not completely align with previous studies showing that men may not inhibit sexual response to rape scenarios (e.g., Malamuth et al., 1986). One possible reason for the low levels of sexual arousal is that the stimuli were short (40 s) in duration. At the same time, the possibility should be considered that the low levels of self-reported sexual arousal we found may reflect a social desirability bias. Similarly, EMG records may benefit from longer baseline responses; accordingly, we encourage researchers to consider longer baselines in future studies. Finally, emotional responses to a rape scenario could be contrasted with mainstream, consensual erotica.

The current study contributes to our understanding of men’s subjective sexual and affective responses to depictions of sexual violence. Additionally, the current findings point to a role of personality and trait sexual inhibition proneness and thus add to the understanding of individual differences in the emotional appraisal of rape. Personality tendencies seem to be consistently involved in sexual violence dynamics, which further stresses their role as key factors.

## Note

1 A priori power analysis was performed to estimate an adequate sample size using G*Power 3.1.9.2 software. Using the type I error (α) criterion of .05, medium effect size (*f* = .25) for repeated-measures ANOVA analysis (3 repeated measurements), with nonsphericity correction (*ε* = 1.00) and anticipated power level (.80), the minimum required sample size was calculated to be 28.

## References

[CIT0001] Anderson, I., & Quinn, A. (2009). Gender differences in medical students’ attitudes towards male and female rape victims. *Psychology, Health & Medicine*, *14*(1), 105–110. 10.1080/13548500802241928 19085317

[CIT0002] Beshers, S., & DiVita, M. (2021). Changes in rape myth acceptance among undergraduates: 2010 to 2017. *Journal of Interpersonal Violence*, *36*(19–20), 9371–9392. 10.1177/0886260519867153 31387449

[CIT0003] Bieneck, S., & Krahé, B. (2011). Blaming the victim and exonerating the perpetrator in cases of rape and robbery: Is there a double standard? *Journal of Interpersonal Violence*, *26*(9), 1785–1797. 10.1177/0886260510372945 20587449

[CIT0004] Boucsein, W. (2012). *Electrodermal activity* (2nd ed.). Springer.

[CIT0005] Cacioppo, J. T., Petty, R. E., Losch, M. E., & Kim, H. S. (1986). Electromyographic activity over facial muscle regions can differentiate the valence and intensity of affective reactions. *Journal of Personality and Social Psychology*, *50*(2), 260–268. 10.1037//0022-3514.50.2.260 3701577

[CIT0006] Carvalho, J., Pereira, R., Barreto, D., & Nobre, P. (2017). The effects of positive versus negative mood states on attentional processes during exposure to erotica. *Archives of Sexual Behavior*, *46*(8), 2495–2504. 10.1007/s10508-016-0875-3 27734171

[CIT0008] Carvalho, J., & Nobre, P. J. (2019). Five-factor model of personality and sexual aggression. *International Journal of Offender Therapy and Comparative Criminology*, *63*(5), 797–814. 10.1177/0306624X13481941 23525179

[CIT0009] Carvalho, J., Quinta-Gomes, A., & Nobre, P. J. (2013). The sexual functioning profile of a non-forensic sample of individuals reporting sexual aggression against women. *The Journal of Sexual Medicine*, *10*(7), 1744–1754. 10.1111/jsm.12188 23668395

[CIT0010] Carvalho, J., & Rosa, P. J. (2020). Gender differences in the emotional response and subjective sexual arousal toward non-consensual sexual intercourse: A pupillometric study. *The Journal of Sexual Medicine*, *17*(10), 1865–1874. 10.1016/j.jsxm.2020.06.018 32723682

[CIT0011] Carvalho, J., Rosa, P. J., & Pereira, B. (2021). Dynamic risk factors characterizing aggressive sexual initiation by female college students. *Journal of Interpersonal Violence*, *36*(5–6), 2455–2477. 10.1177/0886260518760010 29502500

[CIT0012] Colins, O., & Andershed, H. (2016). The Youth Psychopathic Inventory Short Version in a general population sample of emerging adults. *Psychological Assessment*, *28*(5), 449–457. 10.1037/pas0000189 26302107

[CIT0013] Costa, P. T., Jr., & McCrae, R. R. (1992). *Revised NEO Personality Inventory (NEO-PI-R) and NEO Five-Factor Inventory (NEO-FFI): Professional manual*. Psychological Assessment Resources.

[CIT0014] European Union Agency for Fundamental Rights. (2015). *Violence against women: An EU survey*. Publications Office of the European Union.

[CIT0015] Fanti, K. A., Kyranides, M. N., Georgiou, G., Petridou, M., Colins, O. F., Tuvblad, C., & Andershed, H. (2017). Callous-unemotional, impulsive-irresponsible, and grandiose-manipulative traits: Distinct associations with heart rate, skin conductance, and startle responses to violent and erotic scenes. *Psychophysiology*, *54*(5), 663–672. 10.1111/psyp.12837 28169424

[CIT0016] Fanti, K. A., Kyranides, M. N., & Panayiotou, G. (2017). Facial reactions to violent and comedy films: Association with callous–unemotional traits and impulsive aggression. *Cognition & Emotion*, *31*(2), 209–224. 10.1080/02699931.2015.1090958 26469744

[CIT0017] Fanti, K. A., Panayiotou, G., Kyranides, M. N., & Avraamides, M. N. (2016). Startle modulation during violent films: Association with callous–unemotional traits and aggressive behavior. *Motivation and Emotion*, *40*(2), 321–333. 10.1007/s11031-015-9517-7

[CIT0018] Fridlund, A. J., & Cacioppo, J. (1986). Guidelines for human electromyographic research. *Psychophysiology*, *23*(5), 567–589. 10.1111/j.1469-8986.1986.tb00676.x 3809364

[CIT0019] Galinha, I. C., & Pais-Ribeiro, J. L. (2012). Contribuição para o estudo da versão Portuguesa da Positive and Negative Affect Schedule (PANAS): II—Estudo psicométrico [Study of adaptation of the Positive and Negative Affect Schedule to the Portuguese population]. *Análise Psicológica*, *23*(2), 219–227. 10.14417/ap.84

[CIT0020] Hald, G. M., & Malamuth, N. N. (2015). Experimental effects of exposure to pornography: The moderating effect of personality and mediating effect of sexual arousal. *Archives of Sexual Behavior*, *44*(1), 99–109. 10.1007/s10508-014-0291-5 24729134

[CIT0021] Hansen, N. B., Nielsen, L. H., Bramsen, R. H., Ingemann-Hansen, O., & Elklit, A. (2015). Attrition in Danish rape reported crimes. *Journal of Police and Criminal Psychology*, *30*(4), 221–228. 10.1007/s11896-014-9159-9

[CIT0022] Janssen, E., Vorst, H., Finn, P., & Bancroft, J. (2002). The Sexual Inhibition (SIS) and Sexual Excitation (SES) Scales: II. Predicting psychophysiological response patterns. *Journal of Sex Research*, *39*(2), 127–132. 10.1080/00224490209552131 12476244

[CIT0023] Jolliffe, D., & Farrington, D. P. (2004). Empathy and offending: A systematic review and meta-analysis. *Aggression and Violent Behavior*, *9*(5), 441–476. 10.1016/j.avb.2003.03.001

[CIT0024] Jonason, P. K., Li, N. P., Webster, G. D., & Schmitt, D. P. (2009). The dark triad: Facilitating a short-term mating strategy in men. *European Journal of Personality*, *23*(1), 5–18. 10.1002/per.698

[CIT0025] Jordan, J. (2011). Here we go round the review-go-round: Rape investigation and prosecution-Are things getting worse not better? *Journal of Sexual Aggression*, *17*(3), 234–249. 10.1080/13552600.2011.613278

[CIT0026] Kassing, L. R., & Prieto, L. R. (2003). The rape myth and blame-based beliefs of counselorsin-training toward male victims of rape. *Journal of Counseling & Development*, *81*(4), 455–461. 10.1002/j.1556-6678.2003.tb00272.x

[CIT0027] Knight, R. A., & Guay, J. P. (2018). The role of psychopathy in sexual coercion against women: An update and expansion. In C. J. Patrick (Ed.), *Handbook of psychopathy* (Vol. II, pp. 662–681). The Guilford Press.

[CIT0028] Lang, P. J., Simons, R. F., & Balaban, M. T. (1997). *Attention and orienting: Sensory and motivational processes*. Lawrence Erlbaum Associates Publishers.

[CIT0029] Larsen, J. T., Norris, C. J., & Cacioppo, J. T. (2003). Effects of positive and negative affect on electromyographic activity over zygomaticus major and corrugator supercilii. *Psychophysiology*, *40*(5), 776–785. 10.1111/1469-8986.00078 14696731

[CIT0030] Lerner, J. S., Li, Y., Valdesolo, P., & Kassam, K. S. (2015). Emotion and decision-making. *Annual Review of Psychology*, *66*, 799–823. 10.1146/annurev-psych-010213-115043 25251484

[CIT0031] Lonsway, K. A., & Fitzgerald, L. F. (1994). Rape myths: In review. *Psychology of Women Quarterly*, *18*(2), 133–164. 10.1111/j.1471-6402.1994.tb00448.x

[CIT0032] Lykken, D. T., & Venables, P. H. (1971). Direct measurement of skin conductance-A proposal for standardization. *Psychophysiology*, *8*(5)*1971*, 656–672. 10.1111/j.1469-8986.1971.tb00501.x 5116830

[CIT0033] Magalhães, E., Salgueira, A., Gonzalez, A. J., Costa, J. J., Costa, M. J., Costa, P., & Lima, M. (2013). NEO-FFI: Psychometric properties of a Short Personality Inventory. *Psicologia, Reflexão e Crítica*, 4, 242-257.

[CIT0034] Malamuth, N. M., & Check, J. V. P. (1980). Penile tumescence and perceptual responses to rape as function of victims’ perceived reactions. *Journal of Applied Social Psychology*, *10*(6), 528–547. 10.1111/j.1559-1816.1980.tb00730.x

[CIT0035] Malamuth, N. M., Check, J. V. P., & Briere, J. (1986). Sexual arousal in response to aggression: Ideological, aggressive and sexual correlates. *Journal of Personality and Social Psychology*, *50*(2), 330–340. 10.1037//0022-3514.50.2.330 3701580

[CIT0037] Martins, S., Machado, C., Abrunhosa, R., & Manita, C. (2012). Escala de Crenças sobre a Violência Sexual [Rape Myths Scale]. *Análise Psicológica*, *30*(1/2), 177–191. 10.14417/ap.546

[CIT0038] Mirabella, G. (2018). The weight of emotions in decision-making: How fearful and happy facial stimuli modulate action readiness of goal-directed actions. *Frontiers in Psychology*, *9*, 1334. 10.3389/fpsyg.2018.01334 30116211 PMC6083043

[CIT0039] Morabito, M. S., Pattavina, A., & Williams, L. M. (2019). It all just piles up: Challenges to victim credibility accumulate to influence sexual assault case processing. *Journal of Interpersonal Violence*, *34*(15), 3151–3170. 10.1177/0886260516669164 27655867

[CIT0040] Pechorro, P., Andershed, H., Ray, J. V., Maroco, J., & Gonçalves, R. A. (2015). Validation of the Youth Psychopathic Traits Inventory and Youth Psychopathic Traits Inventory – Short Version among incarcerated juvenile delinquents. *Journal of Psychopathology and Behavioral Assessment*, *37*(4), 576–586. 10.1007/s10862-015-9490-1

[CIT0041] Pechorro, P., Ribeiro da Silva, D., Rijo, D., Gonçalves, R. A., & Andershed, H. (2017). Psychometric properties and measurement invariance of the Youth Psychopathic Traits Inventory – Short Version among Portuguese youth. *Journal of Psychopathology and Behavioral Assessment*, *39*(3), 486–497. 10.1007/s10862-017-9597-7

[CIT0042] Peterson, Z. D., Janssen, E., & Heiman, J. R. (2010). The association between sexual aggression and HIV risk behavior in heterosexual men. *Journal of Interpersonal Violence*, *25*(3), 538–556. 10.1177/0886260509334414 19474034

[CIT0043] Quinta-Gomes, A. L., Janssen, E., Santos-Iglesias, P., Pinto-Gouveia, J., Fonseca, L. M., & Nobre, P. J. (2018). Validation of the Sexual Inhibition and Sexual Excitation Scales (SIS/SES) in Portugal: Assessing gender differences and predictors of sexual functioning. *Archives of Sexual Behavior*, *47*(6), 1721–1732. 10.1007/s10508-017-1137-8 29536260

[CIT0059] Revelle, W. (2022). psych: Procedures for psychological, psychometric, and personality research (2.2.9 ed.) [Computer software manual]. psych. (R package version 2.2.9)

[CIT0060] R Core Team. (2022). R: A language and environment for statistical computing [Computer software manual]. Vienna, Austria. Retrieved from https://www.R-project.org/

[CIT0044] Rosa, P. J., Oliveira, J., Alghazzawi, D., Fardoun, H., & Gamito, P. (2017). Affective and physiological correlates of perception of unimodal and bimodal emotional stimuli. *Psicothema*, *29*(3), 364–369. 28693708 10.7334/psicothema2016.272

[CIT0045] Rosenstein, J. E. (2015). Military sexual assault prevention and male rape myth acceptance. *Military Behavioral Health*, *3*(4), 207–211. 10.1080/21635781.2015.1038404

[CIT0046] Sapolsky, B. S. (1984). Arousal, affect, and the aggression-moderating effect of erotica. In N. M. Malamuth & E. Donnerstein (Eds.), *Pornography and sexual aggression* (pp.85–114). Academic Press.

[CIT0047] Smith, C. A., & Kirby, L. D. (2001). Toward delivering on the promise of appraisal theory. In K. R. Scherer, A. Schorr, & T. Johnstone (Eds.), *Appraisal processes in emotion: Theory, methods and research* (pp. 121–138). Oxford University Press.

[CIT0048] Smith, O., & Skinner, T. (2017). How rape myths are used and challenged in rape and sexual assault trials. *Social & Legal Studies*, *26*(4), 441–466. 10.1177/0964663916680130

[CIT0049] Smith, S. G., Zhang, X., Basile, K. C., Merrick, M. T., Wang, J., Kresnow, M., & Chen, J. (2018). *The National Intimate Partner and Sexual Violence Survey (NISVS): 2015 Data brief – updated release*. National Center for Injury Prevention and Control, Centers for Disease Control and Prevention.

[CIT0050] Tomaka, J., Blascovich, J., Kelsey, R. M., & Leitten, C. L. (1993). Subjective, physiological, and behavioral effects of threat and challenge appraisal. *Journal of Personality and Social Psychology*, *65*(2), 248–260. 10.1037/0022-3514.65.2.248

[CIT0051] Turchik, J. A., & Edwards, K. M. (2012). Myths about male rape: A literature review. *Psychology of Men & Masculinity*, *13*(2), 211–226. 10.1037/a0023207

[CIT0052] Vahedi, L., Stuart, H., Etienne, S., Lee, S., & Bartels, S. A. (2021). Gender-stratified analysis of Haitian perceptions related to sexual abuse and exploitation perpetrated by UN peacekeepers during MINUSTAH. *Sexes*, *2*(2), 216–243. 10.3390/sexes2020019

[CIT0053] Van Baardewijk, Y., Andershed, H., Stegge, H., Nilsson, K., Scholte, E., & Vermeiren, R. (2010). Development and tests of short versions of the Youth Psychopathic Traits Inventory and the Youth Psychopathic Traits Inventory-Child Version. *European Journal of Psychological Assessment*, *26*(2), 122–128. 10.1027/1015-5759/a000017

[CIT0054] Voller, E. K., & Long, P. J. (2010). Sexual assault and rape perpetration by college men: The role of the big five personality traits. *Journal of Interpersonal Violence*, *25*(3), 457–480. 10.1177/0886260509334390 19443734

[CIT0055] Walfield, S. M. (2021). ‘Men cannot be raped’: Correlates of male rape myth acceptance. *Journal of Interpersonal Violence*, *36*(13–14), 6391–6417. 10.1177/0886260518817777 30556453

[CIT0056] Watson, D., Clark, L., & Tellegen, A. (1988). Development and validation of brief measures of positive and negative affect: The PANAS scales. *Journal of Personality and Social Psychology*, *54*(6), 1063–1070. 10.1037//0022-3514.54.6.1063 3397865

[CIT0057] World Health Organization. (2013). *Global and regional estimates of violence against women: Prevalence and health effects of intimate partner violence and non-partner sexual violence*. WHO.

[CIT0058] Zillman, D., Bryant, J., Comisky, P. W., & Medoff, N. J. (1981). Excitation and hedonic valence in the effect of erotica on motivated inter-male aggression. *European Journal of Social Psychology*, *11*(3), 233–252. 10.1002/ejsp.2420110301

